# Preheated acid associated with silane and electric current in the adhesion of the resin cement to ceramic

**DOI:** 10.1590/0103-6440202405641

**Published:** 2024-03-22

**Authors:** Gustavo Belmiro Casaburi, Marcos Henrique Ramos da Silva, Lourenço Correr-Sobrinho, Rafael Leonardo Xediek Consani

**Affiliations:** 1 Piracicaba Dental School, State University of Campinas, SP, Brazil.; 2 PhD Program in Dental Materials, Piracicaba Dental School, State University of Campinas, SP, Brazil.; 3 Piracicaba Dental School, Department of Restorative Dentistry, Dental Materials Division, State University of Campinas, SP, Brazil.; 4 Piracicaba Dental School, Department of Prosthodontics and Periodontology, Complete Denture Division, State University of Campinas, SP, Brazil.

**Keywords:** Ceramic, acid etching, silane, electrical current, adhesive bond

## Abstract

This study verified the effect of the combination of preheated hydrofluoric acid/silane/electric current in the adhesion of the resin cement to ceramic. IPS E.max Press ceramic discs embedded in PVC rigid tubes were divided into four groups associating preheated hydrofluoric acid and silane applied with electrical current (n=10): Ha+S (Heated acid + silane); Ha+S+Ec (Heated acid + silane + electrical current); A+S (Acid + silane) and A+S+Ec (Acid + silano + electrical current). Resin cement/ceramic samples were stored in water at 37°C for 24h. After storage, they were submitted to the microshear test, fracture analysis, and contact angle at 24h or after thermocycling (10,000 cycles/5-55ºC). Bond strength data were evaluated by two-way ANOVA. For comparison between evaluation times (24h or thermocycling) was applied unpaired t-test. A significance post-hoc test of p=0.05 was assumed for analyses and graphs (GraphPad Prism 9.0 software). At 24h, the microshear strength showed similar values between Ha+S, Ha+S+Ec, and A+S+Ec groups, while A+S showed the lowest value with a statistical difference. After thermocycling, Ha+S and Ha+S+Ec were similar, as well as A+S and A+S+Ec. There was a significant difference in all groups comparing 24h (highest value) with after thermocycling (lowest value). Adhesive fracture was predominant in all groups and evaluation times. Ha+S and A+S groups showed higher contact angle values compared to the Ha+S+Ec and A+S+Ec with lower values. In conclusion, the association of preheated hydrofluoric acid/silane applied or not with electric current promoted different microshear strength values, fracture types, and contact angles in the resin cement/ceramic bond.

## Resumo

Este estudo verificou o efeito da combinação ácido fluorídrico pré-aquecido/silano/corrente elétrica na adesão do cimento resinoso à cerâmica. Os discos cerâmicos IPS E.max Press embutidos em tubos rígidos de PVC foram separados em quatro grupos associando ácido fluorídrico pré-aquecido e silano aplicado com corrente elétrica (n=10): Ha+S (ácido aquecido + silano); Ha+S+Ec (Ácido aquecido + silano + corrente elétrica); A+S (Ácido + silano) e A+S+EC (Ácido + silano + corrente elétrica). Amostras de cimento resinoso/cerâmica foram armazenadas em água a 37°C por 24 horas. Após o armazenamento foram submetidas ao ensaio de micro cisalhamento, análise de fratura e ângulo de contato no período de 24 horas ou após termociclagem (10.000 ciclos/5-55ºC). Os dados de resistência de união foram avaliados por ANOVA dois fatores. Para comparação entre os tempos de avaliação (24 horas ou termociclagem) foi aplicado o teste t não pareado. Foi assumida significância de 5% para análises e gráficos (software GraphPad Prism 9.0). Em 24 horas, a resistência ao micro cisalhamento apresentou valores similares entre os grupos Ha+S, Ha+S+Ec e A+S+Ec, enquanto A+S apresentou menor valor com diferença estatística. Após a termociclagem, Ha+S e Ha+S+Ec foram similares, assim como A+S e A+S+Ec. Houve diferença significativa em todos os grupos comparando 24 horas (maior valor) com após termociclagem (menor valor). A fratura adesiva foi predominante em todos os grupos e tempos de avaliação. Os grupos Ha+S e A+S apresentaram maiores valores de ângulos de contato comparados aos grupos Ha+S+Ec e A+S+Ec com valores menores. Em conclusão, a associação ácido fluorídrico pré-aquecido/silano aplicado com corrente elétrica promoveu diferentes valores de resistência ao micro cisalhamento, tipos de fratura e ângulos de contato na adesão do cimento resinoso à cerâmica.

## Introduction

Prosthetic restorations with dental ceramics have increased due to various factors related to rehabilitation protocols, mechanical strength, and mimetic characteristics. The ceramic must be resistant to time and to the morphophysiological impacts of the stomatognathic system. However, adequate mechanical strength also depends on the action of several other factors, such as dentin moisture conditions, surface treatments, adhesive luting techniques, and removal of excess dentin moisture ^1^.

Several techniques have been proposed to increase the bond strength between dental substrate, resin cement, and ceramics with fewer clinical steps, as well as decrease the damage to the hard tissues during tooth preparation. Previous studies showed that the application of adhesive systems with electric-current-assisted increased the surface roughness and wettability of the dentin ^2^ ^,^ ^3^. 

In this context, the application of adhesive systems with a device denominated ElectroBond showed to be an efficient strategy to increase the wettability of the dentin, reducing the contact angle ^4^. A recent study also showed that the silane applied with electrical current promoted different adhesive strength levels, failure modes, and contact angles between resin cement and acid-sensitive ceramic ^5^. In addition, adhesive systems applied with electrical current (25 and 50μA) improved the degree of conversion, reduced the nanoleakage, and increased the dentin infiltration when evaluated after 1 year-water of storage, while both the electrical current intensities promoted bond interface with similar stability levels ^6^. 

Dental ceramics surface treatments aiming to increase the roughness, wettability, and bond strength of the adhesive interface have been proposed in earlier studies ^7^ ^,^ ^8^. Thus, the application of 10% hydrofluoric acid to partially remove the vitreous matrix and promote micro retentions has been associated with silane for surface treatments of dental ceramics to increase the bond strength to the resin cement ^7^. Furthermore, thermocycling and long-term water storage decreased the strength of the adhesive bond, affecting differently this mechanical property. However, the surface roughness caused by abrasive rotary instruments and/or chemical etching with hydrofluoric acid increased the bond strength with different values ^8^. 

Previous results showed significant differences in shear strength for different bonding groups, depending on the condition of the ceramic surface. For the unetched samples, significant differences in the bond strength for all bond protocols were obtained. However, for the conditioned groups there was no difference between silane and silane associated with the dentin adhesive. Porcelain etching significantly increased the strength in all bonding methods and was the main contributor to the obtained values ^9^. 

Aiming to improve the strength of the adhesive bond, a previous study showed that surface treatment is important to bond the ceramic, and should preferably be made with hydrofluoric acid instead of phosphoric acid ^10^. On the other hand, 10% hydrofluoric acid etching at different times or sandblasting with 50µm aluminum oxide particles increased the surface irregularities of the IPS Empress, IPS Empress 2, and Cergogold ceramics. However, similar treatments for In-Ceram Alumina, In-Ceram Zirconia, and Procera did not change the ceramic surface microstructure ^11^. 

Procedures that increase clinical steps should be considered since create negative expectations about working time. However, the application of adhesives with the ElectroBond device reduced the contact angle and increased the wettability of the dentin treated with H₃PO4 or Clearfil SE Bond primer, but not when the dentin was treated with the Prompt-L-Pop adhesive system ^4^. 

Luting dental ceramics with resin cement resulted in significant bonding strengthening, probably impacting clinical performance. The bond strengthening is dependent on the creation of a resin-ceramic hybrid layer sensitive to luting variables and clinical application techniques. However, the short-term water immersion resulted in significant degradation of the characteristics of the hybrid layer formed ^12^. Therefore, failure stresses in biaxially loaded resin cement/ceramic bilayers will impact the obtained strength magnitude that accounts for the mismatch between the elastic constants of dissimilar materials ^13^. 

The main emphasis would be also related to the use of silanes in adhesive dentistry. The interpretation of several results is based on controversial observations that can lead to several doubts. However, some clinical results showed that the silanes play a significant role in the bond process and there are different protocols for the bond mechanism between silanes and tooth substrates ^14^. In this context, a previous study considered that the etching of the ceramic surface with hydrofluoric acid is essential for the adhesive protocol ^10^. In addition, the etching with 9% hydrofluoric acid heated to 70°C resulted in significantly higher surface roughness and improved bond strength of the lithium-disilicate glass-ceramic compared to the etching with hydrofluoric acid at room temperature ^15^.

Based on these considerations, it is believed that the association of heated hydrofluoric acid/silane applied with electrical current would be an interesting and timely study in relation to the bond strength of the resin cement to dental ceramic. This study aimed to evaluate the effect of the association of preheated hydrofluoric acid/silane applied with electrical current on the microshear strength, fracture analysis, and contact angle of the resin cement/ceramic bond. The hypothesis of the study was that the association of preheated hydrofluoric acid/silane applied with electrical current would increase the bond strength, and decrease the number of adhesive failures and the contact angle.

## Materials and methods

### Study design

**Figure 1:**
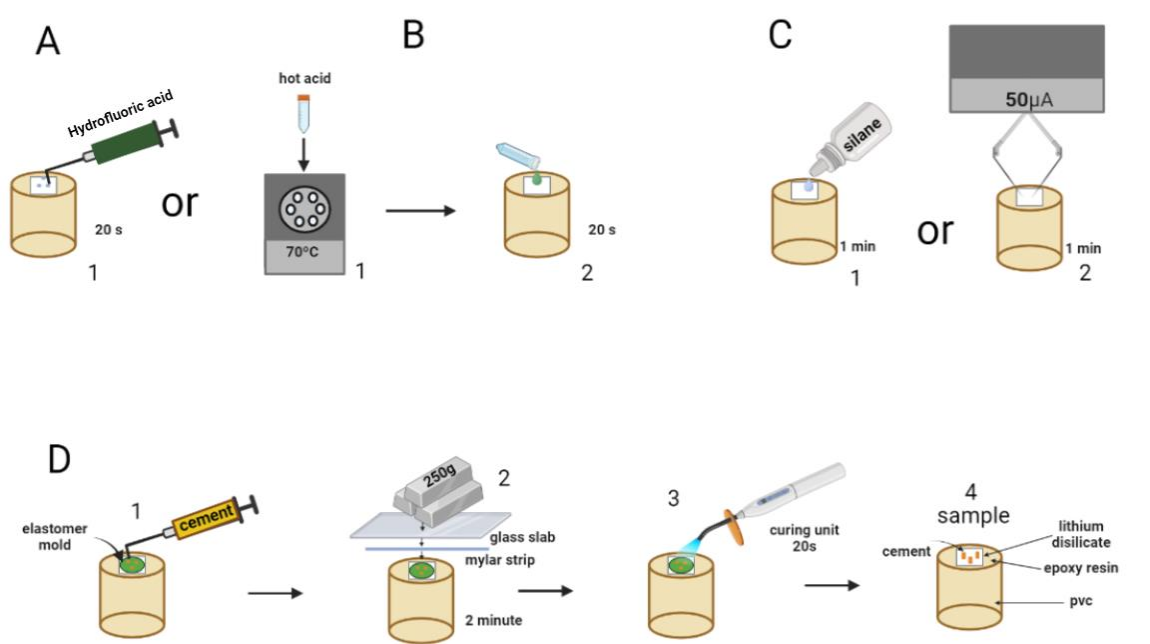
Design of the experimental study: Hydrofluoric acid application (A1), Hydrofluoric acid heated at 70ºC (B1, B2). Silane application (C1); Silane applied with electrical current (C2); Resin cement application (D1); Polyester strip + glass slab + a static load of 250g for 2 min (D2); Photo activation (C3); Sample for microshear strength test (C4).

The study was carried out in vitro according to ISO 4049 (2009) and ISO 6872 (2015) to evaluate polymeric and ceramic dental materials, respectively. IPS E.max Press ceramic discs were divided into four experimental groups (n=10): Ha+S (Heated acid + silane); Ha+S+Ec (Heated acid + silane + electrical current); A+S (Acid + silane) and A+S+Ec (Acid + silano + electrical current). Samples with resin cement bonded to ceramic were submitted to the microshear test, fracture analysis, and contact angle, considering the factors of surface treatment and aging (24h or after thermocycling). The electrical device alternative to Eletrobond and the application method used in the study were based on previous study ^12^. Figure 1 shows the design of the experimental study.

### Preparation of ceramic samples

Ceramic discs were made using the lost wax technique. Duralay acrylic resin patterns (Reliance, Sao Paulo, SP, Brazil) with 2.5mm in thickness x 20mm in diameter were individually embedded in IPS PressVest Speed investment (Ivoclar Vivadent, Barueri, SP, Brazil) and heated in oven to eliminate the wax pattern. IPS E.max Press ceramic (Ivoclar Vivadent) was injected into the mold according to the manufacturer's recommendations. The ceramic discs were polished with abrasive tips and rubbers until reaching a thickness of 2.0 mm and checked with a digital caliper (Mitutoyo, Tokyo, Japan).

 Following, the discs were individually embedded in rigid PVC tubes (25mm in diameter x 30mm in height) with chemically activated acrylic resin (VIPI, Pirassununga, SP, Brazil). The bonding surface of the discs was polished with sandpaper (grit 400 and 600; Norton, Sao Paulo, SP, Brazil) in a polisher (APL4; Arotec, Cotia, SP, Brazil), blasted with aluminum oxide particles (50µm) with a pressure of 3 bar for 30s, cleaned in ultrasound (MaxiClean 750; Unique, Indaiatuba, SP, Brazil) and dried with air jets.

### Ceramic surface treatment

Sample surface from the Ha+S and Ha+S+Ec groups (24h or after thermal cycling) was etched with 1mL of 5% hydrofluoric acid (Maquira Dental Group, Maringa, PR, Brazil) preheated to 70°C (Thermosmart, Cap-Lab, Sao Paulo, SP, Brazil) for 20s, ultrasonic washed for 5min and air dry for 30s. In the same conditions, the samples from A+S and A+S+Ec (24h or after thermocycling) groups were conditioned with 5% hydrofluoric acid at room temperature.

A silane layer (RelyX Ceramic Primer; 3M ESPE) was applied in the samples of the Ha+S and A+S groups with a microbrush for 15s, dried with a light jet of air, and stored at room temperature for 45s. In the samples from the Ha+S+Ec and A+S+Ec groups, the silane was applied with the metallic tip of the device generator of electrical current, dried with a light jet of air, and stored at room temperature for 45s.

A polyvinylsiloxane matrix (12 mm in diameter x 2mm in thickness) containing three holes (4mm in diameter x 2mm in thickness) configured as an equilateral triangle (sides = 1.5mm) was placed on the bond surface of the ceramic discs embedded in the rigid PVC tubes. Only one author made the resin cement discs on the ceramic surface. The resin cement RelyX U200 (3M ESPE) was sequentially inserted into the silicone matrix holes. A polyester strip was placed over the filled matrix overlapped by a glass slab, and a static load of 250g was applied to the assembly for 2 min. After removal of the static load, glass slab, and polyester strip, the resin cement discs were individually photoactivated with an irradiance of 1,200 mW/cm2 (Bluephase G2, Ivoclar Vivadent, Liechtenstein) for 20s. After, the silicone matrix was removed using a scalpel.

The resin cement cylinders bonded to the ceramic were evaluated using an optical microscope (Olympus, Tokyo Japan) with a 40x magnification, replacing the samples with any structural defect. After being stored in water in an oven at 37ºC for 24h, the samples were divided into groups for evaluation at 24h (control) or after 10,000 thermocycles (MSCT-3e, Elquip, Sao Carlos, SP, Brazil) each cycle of 30s and transfer time of 5s in vat with temperatures of 5 and 55ºC.

### Strength test (µSBS)

The PVC tube containing the cement/ceramic samples was placed in a device adapted to the testing machine. A stainless steel wire (0.2 mm in diameter) was fitted around the cylinder at the bond interface. The bond microshear test was performed in the universal machine (Instron; Model 4411, Canton, MA, USA) with a 50N load cell at a speed of 1.0 mm/min until failure. Data in Newton were converted into MPa and the mean value of three discs was considered the µSBS of each sample. The cylinder cross-sectional area (mm^2^) was used to calculate the strength value, using the formula: MPa = N/mm², where MPa = Fracture strength (MPa); N = Maximum force at the time of fracture, and mm² = sample cross-section.

### Fracture pattern analysis

Sample fractures were analyzed according to the failure mode and classified as adhesive, cohesive in resin cement, cohesive in ceramic, or mixed using a measuring microscope (STM; Olympus, Tokyo, Japan) with 40x magnification. 

### Scanning electron microscopy analysis

A representative sample of each group coated with gold by sputtering (Sputter Coater SCD-050; Balzers, Jundiaí, SP, Brazil) under conditions of 120s, 40µA, and 10nm-thickness was analyzed with scanning electron microscope (JEOL, JSM-5600LV, Tokyo, Japan), obtaining images at 200 and 500x magnification. 

### Contact angle

A sample from each group (n=10) was evaluated in a goniometer with coupled software (Digidrop Contact Angle Meter; GBX, Bourg de Peage, France) using a water drop method (3µL) on the sample surface. The contact angle captured by the Charge-Coupled camera was evaluated by the GBC DIGIDROPTM software (GBX, Bourg de Péage, France) based on the concept that the smaller the contact angle, the greater the substrate wettability. The average of the values of the right and left angles was considered the contact angle of the sample.

### Statistical analysis

The bond strength values of the groups obtained in 24h or submitted to thermocycling were evaluated for normality by the Shapiro-Wilk test and homoscedasticity of variances by the Levene test. As the results showed normal distribution and homogeneous variance, the two-way ANOVA test was applied in both aging periods (p<0.05). The unpaired t-test was applied for comparison between the aging groups (24h and after thermocycling). For all analyses, the post-hoc test of p=0.05 was assumed. The analyses and graphs were obtained using GraphPad Prism 9.0 software.

## Results

### Strength test (µSBS)

**Figure 2:**
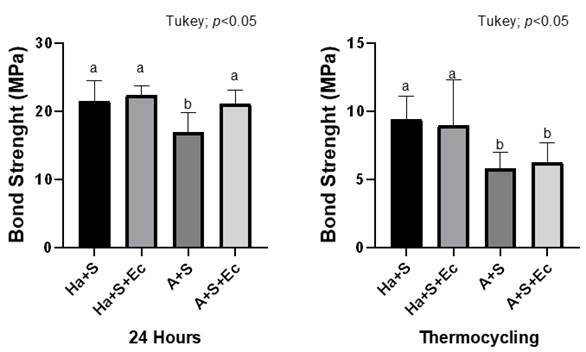
Graphic illustration of the microshear strength values at 24h and after thermocycling.

Table 1 and Figure 2 show the microshear strength test values. Strength values were similar between Ha+S, Ha+S+Ec, and A+S groups at 24h. The A+S group with the lowest value differed from the others. After thermocycling, the Ha+S and Ha+S+Ec groups were similar with higher values, and A+S and A+S+Ec groups were similar with lower values. 

**Table 1:** Microshear strength (mean ± standard deviation, MPa) at 24h and after thermocycling.

	24 Hours	Thermocycling
Ha+S	21.44 (1.7) a	9.39 (3.2) a
Ha+S+Ec	22.33 (3.3) a	8.96 (4.0) a
A+S	16.95 (1.2) b	5.77(1.78) b
A+S+Ec	21.04 (1.4) a	6.26 (2.0) b

Means followed by equal letters in each column do not differ statistically by Tukey's test (5%).

**Figure. 3:**
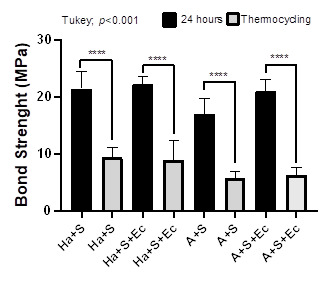
Graphic illustration of the microshear strength values comparing 24h and after thermocycling evaluations.

Figure 3 shows the microshear strength values comparing the 24h and after thermocycling evaluations. There was a statistically significant difference in all groups when 24h (highest value) was compared to after thermocycling (lowest value). All the factors (surface treatment and aging periods) and interaction (surface treatment x aging periods) were significant (p<0.001).

### Fracture pattern analysis

Table 2 shows the failure mode values at 24h and after thermocycling (%). At 24h, the predominant fracture was adhesive in all groups. Cohesive fracture in resin cement occurred for the Ha+S, Ha+S+Ec, and A+S+Ec groups. After thermocycling, adhesive fracture was predominant in all groups. Cohesive fracture in resin cement occurred for Ha+S, Ha+S+Ec, and A+S+Ec groups. There were no mixed fractures or cohesive in ceramic for all groups and evaluation periods.

**Table 2:** Fracture pattern at 24h and after thermocycling (%).

24 hours				
	Mixed	Adhesive	Cohesive*	Cohesive^#^
Ha+S	0.0%	93.3%	0.0%	6.7%
Ha+S+Ec	0.0%	76.7%	0.0%	13.3%
A+S	0.0%	100%	0.0%	0.0%
A+S+Ec	0.0%	0.0%	0.0%	10.0%
Thermocycling				
	Mixed	Adhesive	Cohesive*	Cohesive^#^
Ha+S	0.0%	98.6%	0.0%	1.3%
Ha+S+Ec	0.0%	92.4%	0.0%	7.6%
A+S	0.0%	100%	0.0%	0.0%
A+S+Ec	0.0%	98.2%	0.0%	1.8%

* Cohesive in ceramic; ^#^Cohesive in resin cement.

### Scanning electron microscopy analysis

**Figure 4:**
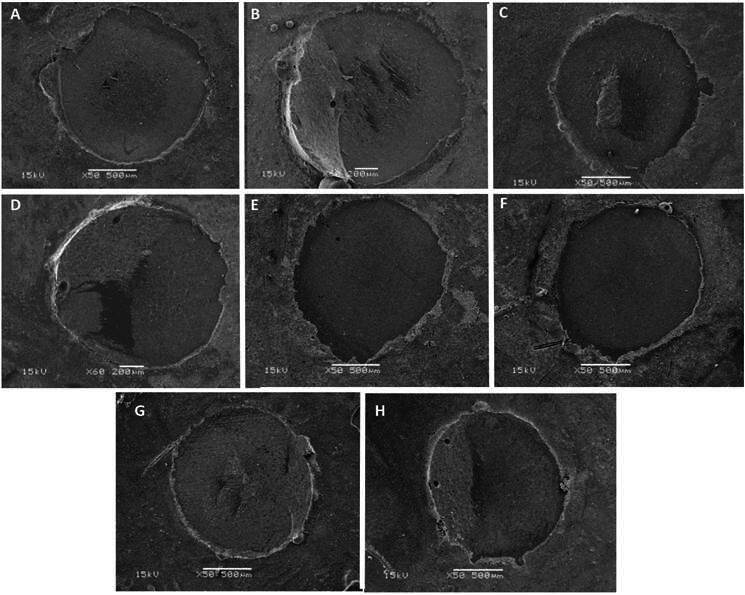
Fracture pattern: Ha+S (A, B - Adhesive and cohesive in resin cement), Ha+S+Ec (C, D - Adhesive and cohesive in resin cement), A+S (E, F - Adhesive) and A+S+Ec (G, H - Adhesive and cohesive in resin cement). Micrographs magnified at 50 and 60x.

Figure 4 shows representative micrographs of the failure patterns obtained in SEM with 50 and 60x magnifications.

### Contact angle

**Figure 5:**
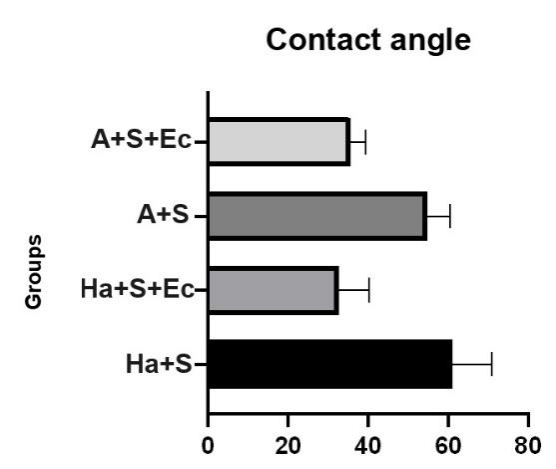
Graphic illustration of the contact angle values (º).

Figure 5 shows the contact angle values. The Ha+S and A+S groups presented higher values compared to the Ha+S+Ec and A+S+Ec groups with lower values.

## Discussion

The present study was developed based on previous investigations claiming that the silane applied with electrical current ^5^, as well as the ceramic heating combined with preheating and concentrations of the hydrofluoric acid ^16^, can improve the bond strength of the resin cement to ceramic. Thus, this investigation verified the effect of the association preheated hydrofluoric acid/silane applied with electrical current (50µA) on the bond strength, failure types, and contact angle of the resin cement/acid-sensitive ceramic bond evaluated at 24h or after thermocycling.

Table 1 and Figure 2 showed that at 24h the Ha+S (21.44 MPa), Ha+S+Ec (22.33 MPa), and A+S+Ec (21.04 MPa) groups promoted µSBS with statistically similar values. The lowest mean of µSBS was found for the A+S group (16.95 MPa) with statistical difference compared to the other groups. After thermocycling, the A+S (5.77 MPa) and A+S+Ec (6.26 MPa) groups showed lower values and statistical differences compared to the Ha+S (9.39 MPa) and Ha+S+Ec (8.96 MPa) groups with greater values. The µSBS values between 24h (greater) and after thermocycling (smaller) showed significant differences in all groups (Figure 3). Therefore, the hypothesis of the study that the association of preheated hydrofluoric acid/silane applied with electrical current would increase the bond strength was not confirmed.

However, the analyzed adhesive protocols can be considered promising, since the preheated acid with or without electrical current or acid at room temperature with electrical current showed higher µSBS values. In addition, the samples with preheated acid with or without electrical current showed greater and similar values, and those samples etched with acid at room temperature with and without electrical current showed smaller and similar values. Previous work-related that the etching with 9% hydrofluoric acid etching heated to 70°C resulted in significantly higher surface roughness and improved bond strength of the lithium-disilicate glass-ceramic compared to hydrofluoric acid etching at room temperature ^15^. However, the cited study did not verify the effect of preheated hydrofluoric acid associated with silane applied with electrical current in the adhesive bond of the ceramic. 

 An investigation evaluating only the effect of preheated silane showed that the bond strength of resin cement to feldspar ceramic treated with hydrofluoric acid+silane+Panavia F2.0 was similar to the same treatment with silane preheated in the oven at 100°C/2min. However, both treatments showed higher bond strength values than the associations without hydrofluoric acid treatment ^17^. 

On the other hand, heat treatments for the pre-hydrolyzed silane, either in a furnace or with the application of hot air, cannot replace the use of hydrofluoric acid gel for the adhesion of resin cement to feldspathic ceramic. When bond strength and incidence of pre-test failures were considered in the study, the thermal treatment in furnage showed the second-best results after the control group, being considerably better than the heated air application ^18^. In addition, micromechanical roughening of the ceramic surface performed by air abrasion with Al_2_O_3_ particles, and silane heat treatment with constant 120°C airflow applied for 60sec followed by dental adhesive application enhanced the resin-ceramic bond strength ^19^. 

However, it is alleged that the polar movement of molecules using electric current is based on the iontophoresis principle ^4^, a factor that could probably also improve the adhesion of the ceramic by molecular interaction with resin cement. It is possible that the results of the current study can also be interpreted in terms of ionic diffusion and space charge polarization according to Sawada's theory ^20^ since the positive metallic pole of the device supplying the electrical current on the ceramic surface would promote repulsion and/or attraction of the involved molecules in the system. 

It is speculated that resin microleakage may be improved by the attraction of polar monomers by an electrical current or by a change of the dentin surface charges, resulting in better water substitution or evaporation ^2^. On the other hand, the interpretation of various results is based on controversial observations very difficult to explain. Silane reaction mechanisms were not entirely understood, and there exist several theories for bonding mechanisms for silanes and substrates. Nevertheless, the majority of the clinical results pointed to silanes playing a significant role in the adhesion process ^21^.

Hydrofluoric acid has been used in the surface treatment of ceramics to partially expose the matrix crystals, allowing greater roughness, expansion of the area available for bond, and increased surface energy. A previous study showed that the bond strength of lithium disilicate ceramic directly depends on the concentration and etching time of the hydrofluoric acid. For 1% and 2.5% concentrations, the etching times from 40 to 120s increased the μSBS values compared to 20s ^22^. However, previous results showed that the surface-roughening method had the strongest effect on the bond strength, while ceramic selection had the least significant effect. Among the surface-roughening methods, etching was associated with higher bond strength values than either sandblasting or grinding ^23^. 

Improving the morphological characteristics and increasing the bond strength of resin cement to lithium disilicate ceramic has been the purpose of previous investigations. Thus, the study investigating the pre-heating ceramic associated with pre-heating and concentration of the hydrofluoric acid showed that for the control group, a concentration of 5% promoted lower bond strength to microshear compared to 10%. On the other hand, the pre-heated hydrofluoric acid (70ºC) and vitreous ceramic (85ºC) increased the resistance value with a concentration of 5%, promoting results similar to the 10% concentration of not pre-heating samples. In addition, heating at the lowest acid concentration had also a positive effect on the removal of the glassy matrix ^16^. 

Table 2 shows the failure mode values (%). At 24h, the predominant fracture was adhesive in all groups. Cohesive fracture in resin cement occurred for the Ha+S, Ha+S+Ec, and A+S+Ec groups. After thermocycling, adhesive fracture was predominant in all groups. Cohesive fracture in resin cement occurred for Ha+S, Ha+S+Ec, and A+S+Ec groups. There were no mixed or cohesive fractures in ceramic for all groups and evaluation times (Figure 4). Therefore, the hypothesis of the study that the association of preheated hydrofluoric acid/silane applied with electrical current would decrease the number of adhesive failures was not confirmed.

The bond of the resin cement to the dental ceramic occurs by the mechanical imbrication of the cement to ceramic surface irregularities caused by sandblasting with aluminum oxide particles or partial removal of the vitreous matrix conditioned by hydrophosphoric acid, allowing chemical bond promoted by the silane application ^24^. Silane is a bifunctional molecule capable of promoting the bond of the inorganic ceramic with the organic adhesive system, increasing the surface energy and improving the ceramic wettability. For this reason, the silane increased the bond strength in relation to the groups without silane application. However, the most effective surface treatment was with 10% hydrofluoric acid with or not silane application ^7^. 

Figure 5 shows that the contact angles of the Ha+S (59.94º) and A+S (54.81º) groups were greater than for Ha+S+Ec (33.56º) and A+S+Ec (35.60º) groups. Based on this result, the Ha+S+Ec and A+S+Ec groups would indicate the possibility of a lower wettability level compared to the Ha+S+Ec and A+S+Ec, with a greater possibility to increase the wettability of the ceramic due to electrical current application. Therefore, the hypothesis of the study that the association of preheated hydrofluoric acid/silane applied with electrical current would decrease the contact angle was confirmed.

In the present study, the smallest contact angles were obtained with both preheated hydrofluoric acid at room temperature and associated with the electrical current application. This result is in agreement with the positive effect of the electrical current showing that the hydrofluoric acid combined with silane applied with electrical current promoted a lower contact angle in the group without acid/with electrical current and a greater angle in the group with acid/without electrical current ^5^. 

Therefore, studies should be carried out involving different adhesive protocols and facilitator materials of the bond to the hard tooth tissues. These studies should investigate other effects of the electrical current and dental materials preheated in the resin cement/ceramic bond for indirect dental restorations, as the cohesive strength of the preheated resin cement. In addition, the increase of information regarding the effect of preheated materials in the resin cement bond would contribute to understanding the results of these study. The lack of this information can be considered as a limitation of the study. 

## Conclusion

The following conclusions can be considered: 1) The association of the preheated hydrofluoric acid with silane applied with or without electrical current promoted different microshear strength values. 2) The thermocycling decreased the bond strength values. 3) This association promoted different fracture modes and contact angles in both evaluation periods.
